# Supplementary material for: Parasitic wasps avoid ant-protected hemipteran hosts via the detection of ant cuticular hydrocarbons
Source: Proc Biol Sci. 2021 Jan 6;288(1942):20201684. doi: 10.1098/rspb.2020.1684 (PMC7892424; doi:10.1098/rspb.2020.1684)
Supplement: Layout of the experimental setup to obtain mealybug colonies attended by ants ;GC/MS chromatograms of pentane extracts of Teflon-coated bridges employed by ants [file rspb20201684supp1.docx]

**SUPPORTING INFORMATION**

**Figure S1.** Layout of the experimental setup to obtain mealybug colonies attended by ants from (A) queenless ant nests in the laboratory, and (B) field queenright ant nests.

**Figure S2.** GC/MS chromatograms of pentane extracts of Teflon-coated bridges employed by (A) queenless ants during foraging, and (B) control bridges. Labels on peaks represent retention times that are in accordance with compounds in Table 1. The previous part of the chromatograms, from 0 to 27 min, is not shown because specific compounds were not detected during this period.

A


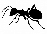

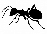

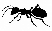

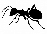

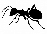

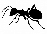

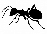

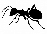

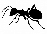

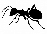

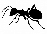

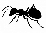

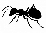

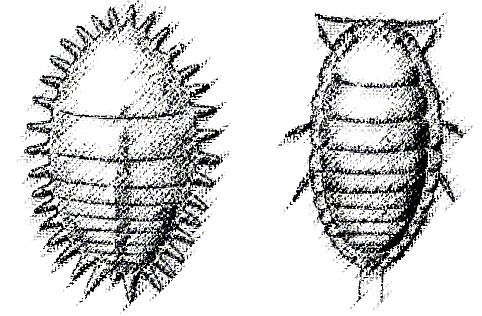

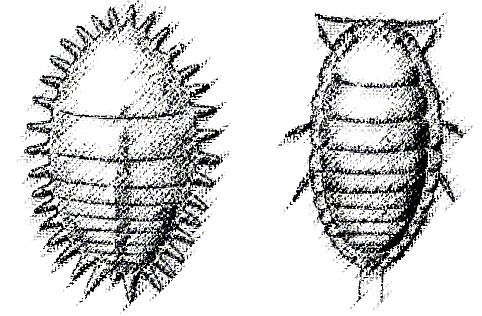

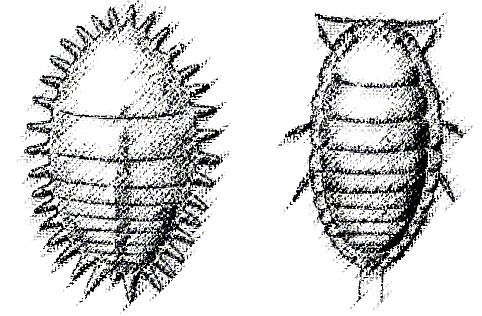

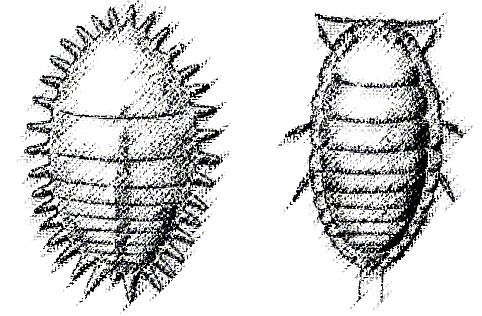

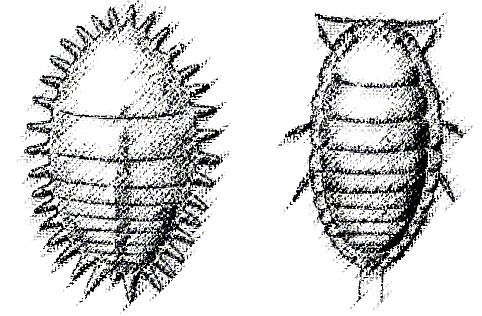

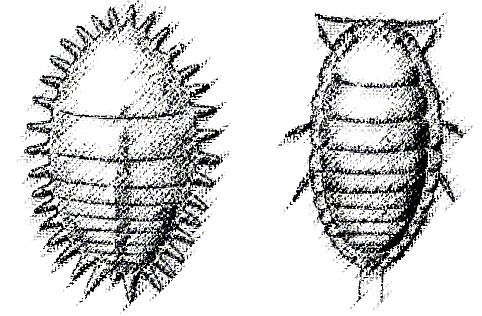

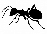

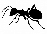

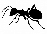

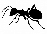

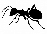


Glass tube with water

Metal bridge connecting ant nest and mealybug colony

Mealybug colony on potato sprout


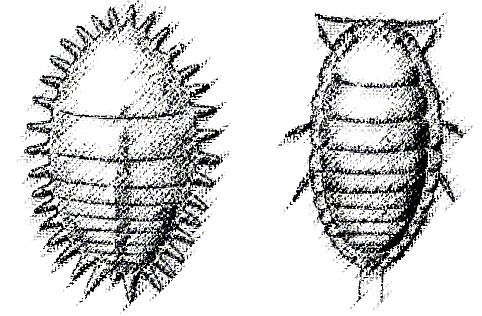

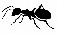

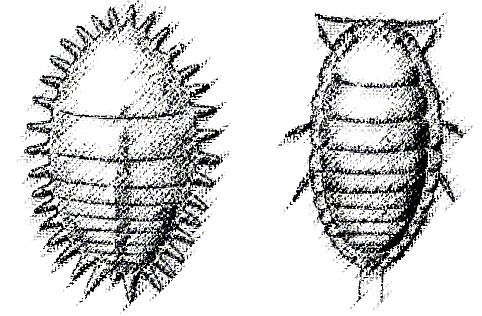

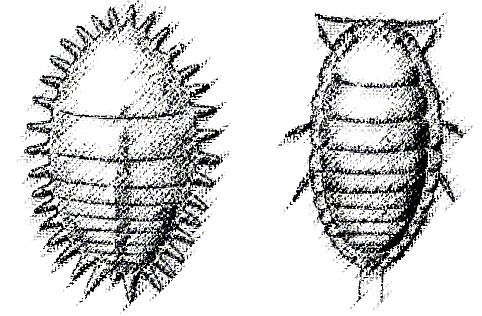

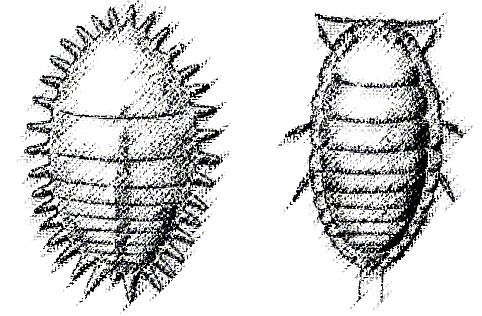

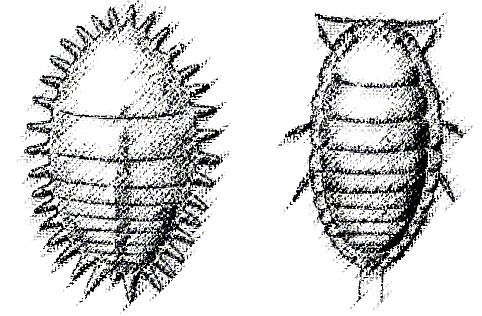

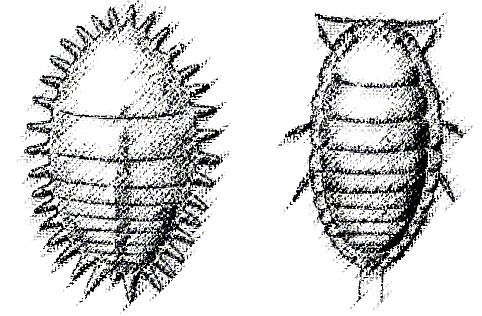

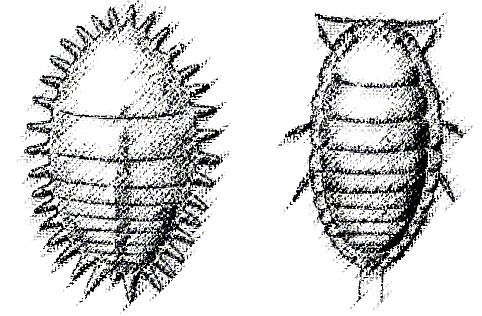

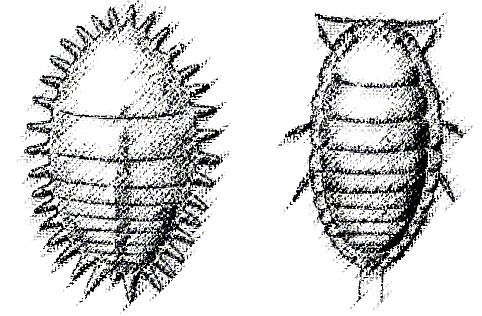

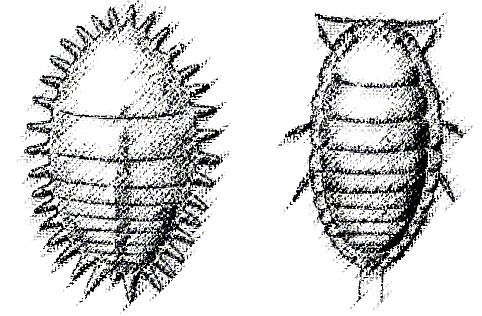

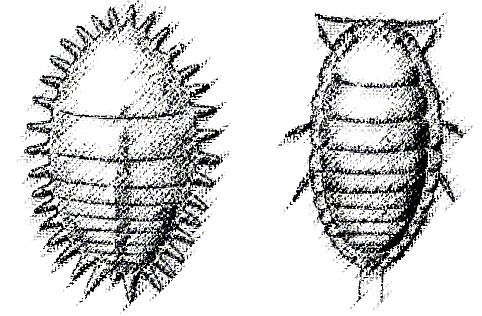

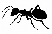

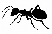

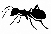

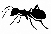

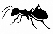

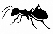

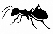

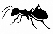


Mealybug colony on bean pod

Holes allowing foraging ants to tend mealybug colonies

**Figure S1.** Layout of the experimental setup to obtain mealybug colonies attended by ants from (A) queenless ant nests in the laboratory, and (B) field queenright ant nests.

B

**
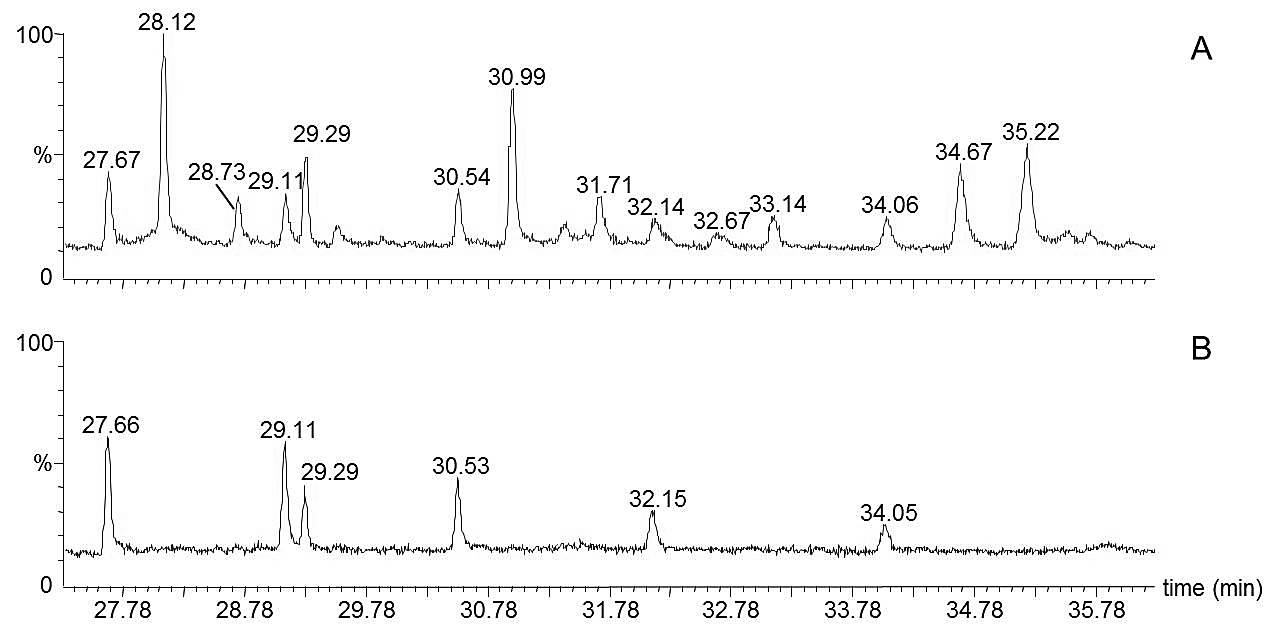
**

**Figure S2**. GC/MS chromatograms of pentane extracts of Teflon-coated bridges employed by (A) queenless ants during foraging, and (B) control bridges. Labels on peaks represent retention times that are in accordance with compounds in Table 1. The previous part of the chromatograms, from 0 to 27 min, is not shown because specific compounds were not detected during this period.
